# Trade and health in Samoa: views from the insiders

**DOI:** 10.1186/1471-2458-14-309

**Published:** 2014-04-04

**Authors:** Jacinta Fa’alili-Fidow, Judith McCool, Teuila Percival

**Affiliations:** 1 Pacific Health, School of Population Health, Faculty of Medical and Health Science, University of Auckland, Private Bag, 92019, Auckland 1142, New Zealand; 2 Social and Community Health, School of Population Health, Faculty of Medical and Health Science, University of Auckland, Private Bag, 92019, Auckland 1142, New Zealand

**Keywords:** Trade, Pacific, Samoa, Non-communicable disease

## Abstract

**Background:**

The purpose of this paper is to portray the views of key stakeholders on the potential impacts of Samoa’s free trade negotiations and agreements, on health and wellbeing in Samoa.

**Methods:**

A series of key informant interviews were undertaken with identified stakeholders during June and July, 2011. Interviews were conducted using a semi-structured interview protocol. They were conducted in–person, in New Zealand and in Samoa.

**Results:**

Despite potential health and wellbeing gains arising from trade activities (employment, increase in income, health innovations and empowerment of women), key stakeholders expressed a growing concern about the effect of trade on the population’s health, nutrition and the rates of non-communicable diseases. Unease about compromising the national policies due to international regulations was also conveyed. Business and trade representatives however, believed that trade benefits outweighed any health and wellbeing risks to the population of Samoa.

**Conclusion:**

Further investigation, using new methodologies are required to determine both the opportunities and threats for trade as a mechanism to improve the health of Samoa’s population.

## Background

As a developing Pacific nation, Samoa has long viewed trade as a necessary and inevitable development strategy to build the national’s economy and reduce poverty [1]. This ideology is consistent with the global free trade movement, it favours a stronger focus on trade as a mechanism to increase country and individual wealth [2]. Our paper presents an analysis of the views of a sample of Samoan and New Zealand trade, public health and government official’s views on the potential impacts of free trade agreements on health in Samoa.

Global free trade agreements have attracted much criticism for their potential to undermine individual access to those key determinants of health (healthy food choices, access to affordable medicine). Friel and colleagues provide a timely critical reflection on the impact of globalised trade on low and middle income countries and their access to affordable nutritious food. Imported foods, although a necessary supplement may undercut local food suppliers of healthy food, flooding the market with food typically higher in salt, fats and sugars [3]. The core of this argument is that during times of global food insecurity, the available affordable and acceptable food options become the true measure of development and trade being the key element of this debate [3]. As a member of the Pacific Islands Forum, Samoa, like the other Pacific Islands Countries (PICs) is a member of PICTA (Pacific Islands Trade Agreement) which is noted as a ‘stepping stone’ to the trade liberalisation agreement with New Zealand and Australia. The Pacific Islands are presently negotiating two key free trade agreements: Pacific Agreement on Closer Economic Relations (PACER) and PACER Plus [4]. At present, PACER Plus, is a framework agreement (non-legal) that establishes a platform for future trade relations between the PICs, Australia and New Zealand. The Pacific Islands are strongly encouraged by the Pacific Islands Forum secretariat to endorse the continuing benefits of broadening trade partnerships within the region. Although, there is an expectation among the Pacific Forum countries of expanding trade relations there is also an understanding amongst leaders of the need for wider consultation and consideration of the implications of ratifying the PICTA and PACER Plus. During this study, Samoa signed three bilateral free trade agreements with USA, Canada and Uruguay, to meet its prerequisites for World Trade Organisation (WTO) membership, and on the 17^th^ of December 2011, it achieved WTO accession status [5].

The processes and concessions required to liberalize trading relationships under the WTO is fraught, and can be intimidating for small island developing countries. Wallis [6] describes the experience for Tonga, concluding that despite the government’s willingness to boost Tonga’s potential via WTO accession, the process was decidedly “unfriendly” and has not been wholly beneficial [6]. Thow and colleagues reviewed Samoa’s changes in trade policies. The impact it has had on food imports and food availability reinforces the impact that local policies are likely to have, even after taking into consideration other social changes [7].

Apprehension amongst the health sector about trade agreements and their implications on public health reflects an inherent lack of trust in a process that lacks transparency and that are legally binding. Such arrangements bind countries to complex long term and inflexible agreements that curtails the government’s capacity to respond with a public policy response [8]. Protecting and promoting population health is still considered a primary government responsibility, yet empirical evidence of this relationship between international trade agreements and health outcomes remains scant [9]. Chronic non-communicable diseases, environmental vulnerability, access to pharmaceuticals and the trade of services, are at the forefront of the concerns expressed to date by health advocates [10]. Ensuring trading negotiators in the Pacific, including Samoa are fully aware of these health implications is vital to protecting populations from inadvertent harm [11]. Our brief report provides an insight on the benefits and challenges of trade liberalization for the Independent State of Samoa.

## Methods

A qualitative study was conducted using a key informant interview method. Key stakeholders working in the areas of government, commerce and/or health in Samoa and New Zealand were identified through professional networks. All interviews were undertaken during June and July 2011 and were conducted using a semi-structured interview protocol in New Zealand, and or, in Samoa. Interviews lasted up to one hour and were audio recorded and abstracted for analysis.

Key informant interviews were conducted to provide primary data exploring both the diversity and similarity of perception around trade, trade negotiations and their longer term impact on the health outcome for Samoa. A list of key stakeholders who held senior leadership roles, their responsibilities and/or experience in the field of trade and/or health was drafted at the start of the study. The list was designed to provide a balanced representation of different sectors, incorporating views from New Zealand and Samoa.

An extensive list of potential questions relating to general trade and health with specific focus on the Pacific region, namely Samoa, was scoped. This list was subsequently culled to 12 key questions which explored the stakeholder’s views. Questions focused on the strategic trade issues, PACER Plus, the effects of trade on health, the role of regional and international organisations in monitoring trade activities and promotion of positive effects of trade negotiations/activities. *Trade as part of a broad economic strategy* for Samoa (e.g. There is a view that trade liberalization is ‘inevitable’ and that sheltered economies need to reform otherwise they will struggle to survive. Do you agree or disagree with this, and why?); *Trade as income* (e.g. What are the benefits for Samoa of signing up to a FTA and how can those benefits be experienced by most, if not all of the population?); *Trade as a health measure* (e.g. Do you think there could be a link between FTAs and downstream health effects, particularly for children)?; *New Zealand’s role as a trade partner* (e.g. Given traditional FTA approaches, what specific roles or responsibilities do you think NZ has to Pacific countries such as Samoa to ensure that FTAs are in the best interests of their economies and populations? *And regional and international organisations* (e.g. Are there any regional and/or international organisations who you think are key players in ensuring that FTAs are beneficial (particularly from a health perspective) for Samoa?

This study was granted ethics approval from the University of Auckland Human Participants Ethics Committee (Ref 7613).

Data analysis was conducted through a general inductive analysis. Interview data was abstracted (key notes were taken from the interview at the time and following the interview on reviewing the audio recordings). Results of the analysis are presented below, but note, these should not be taken as fully representative of other stakeholder’s views in Samoa or the Pacific.

## Results

Seven in-depth interviews were conducted - four with senior leaders in New Zealand (a government department CEO, an NGO CEO, a business and trade CEO and a government Minister) and three were conducted in Samoa (CEO of a regional NGO, a senior business leader and a senior government official). Textual data collected from key informant interviews were analysed using an inductive thematic analysis method and are presented in the table below, reflecting the logical development of dominant themes (Table 1).

**Table 1:** Participants perceptions of how international trade may benefit and pose risks for Samoa’s development

**Perceived trade benefits**	**Perceived trade related risks**
Participation in global movement	Loss of local decision-making/ overturning national health protection policies
WTO accession status – international member and benefits	WTO accession status – top-down approach/lack of consultation
PACER Plus – increased alignment between Samoa and Australia/NZ	PACER Plus - unequal trading platforms i.e. Australia/NZ gain more than Samoa
Increased wealth from business creation	Loss of tariffs – loss of government revenue for essential services e.g. health and education
Improving gender equality – empowerment of women	Increase in inequalities – greater divide between employed and unemployed; urban vs. rural
Increased access to wider range of goods e.g. foods; increase in consumer choice	Nutrition transition – shift in diets resulting from increased availability of high density – nutrient poor foods
Increased trade in services – e.g. increased remittances	Loss of workforce/skills abroad

### Trade and trade liberalization: self-determination versus inevitable and swift transition

Trade liberalization as prescribed in theory - to enable or encourage Samoa to have an *active participation* in the global economy – was perceived as an inescapable outcome for Samoa. Opinions about how this participation in trade might be expressed varied across the stakeholders; a few believed that Samoa needed to *fully* subscribe to the traditional framework of trade liberalization (one dominated by WTO). Alternatively, other stakeholders expressed the importance of self-determination and of protection measures to be embedded within trade agreements so that they do not restrict local visions and policies. Below, was expressed by one stakeholder.

*“Countries need to choose when and how they will open up their economy. It’s that choice that allows your external trade regime to become part of your economic and social development plan. It’s that flexibility in particular those FTAs remove because they pre-empt choices that future governments may make. Trade policies link to broader policies in ways that can be deeply damaging”. *(CEO 1, NZ)

Samoa’s pursuit of WTO accession incited mixed views from stakeholders. Although not all were able to comment on Samoa’s current accession status, there was a clear distinction between those who work in trade and business sectors who favoured the move (towards free-trade agreements), and others who expressed major concerns about the risks of doing so. The mandate for completing the accession process was described by one stakeholder as a purely “top-down approach” with government asserting the need to achieve all the prerequisites. Others viewed it as a rigorous and therefore appropriate progression (since 1988) and that valuable lessons had been learnt from other countries, in particular Tonga (where they accepted a top tariff of 20% in their accession package).

Those opposing the move (towards WTO accession) voiced concerns about the lack of a full consultation with all relevant groups, the need to make everyone aware of the consequences of WTO membership. Stakeholders discussed the potential challenges that Samoa would face in competing with the bigger players:

*“When a country joins WTO they stand to gain but also they have to free up a lot of their local rules around trade (such as) treating foreign products the same as local…there is a trade imbalance…the volume and range of products that the Pacific has to offer the world is so small, limited both in terms of volume and range……..fledgling markets can’t compete with what’s coming in from China at a fraction of the price”.* (CEO 2, NZ)

### PACER plus

PACER Plus (Pacific Agreement on Closer Economic Relations) negotiations were launched by Australia and New Zealand in August 2009 and they continue to be negotiated as recently as November 2013. Pacer Plus was widely accepted as *the* agreement that would veer from the traditional approach of aggressive bargaining and the protection of Australia and New Zealand’s interests towards more of an economic development platform. One New Zealand Government Minister indicated that the needs of the Pacific are critical in these negotiations:

*“…what we see is for once in a trade negotiation, New Zealand exporters take a distant second to the economic development of Pacific countries… we want to use trade negotiation and aid budget to create jobs and exports for Pacific countries”* (Government Minister, NZ)

In general, the stakeholder’s views on PACER Plus negotiations leaned towards either an opportunistic *or* risky outlook. Those who fell in the former camp felt that New Zealand and Australia would have less to gain through PACER Plus than the Pacific nations, as there are already zero-tariffs on Pacific imports to Australia and New Zealand, both these countries pursue significantly larger markets when compared with the Pacific.

*“Australia and New Zealand don’t really care if we say No to PACER Plus because they are already signing agreements with Asian countries and they are much more lenient, including the labour market….so we ask the question, why would they want to focus on us? It’s up to us to get as much access to these markets as possible – we will have a lot of gains from PACER Plus than costs”.* (Business Consultant, Samoa)

Others were less optimistic about Australia and New Zealand’s motivations and were sceptical of the development rhetoric and the potential for power-plays:

*“There are regional leaders who are concerned about being coerced into economies who are heavily aid-dependent – they have no choice, it’s not a level playing field. The minuses will outweigh the pluses from a small island perspective – clearly New Zealand and Australia stand to gain. They’ve got more goods to trade with greater volumes and ranges of products and services”.* (CEO 2, NZ)

A few were less confident in the process and felt that the current negotiations were redundant due to other factors which needed to be addressed first, for example the coup in the Fijian government:

*“Realistically it won’t be in place for another 10 years – you can’t sign without Fiji and they’re in suspension”.* (CEO 3, NZ)

### Trade as essential for achieving development goals?

Stakeholders generally felt that while the exporting of products was a potential area of growth and income generation for Samoa, it would require technical and capacity building investment in order to harness the benefits of trade relationships. The benefits of investing in Samoa’s export trade development were described by one stakeholder as an example of a successful exporting venture:

*“For me the absolute model of good development assistance is the Body Shop project on the other side of Apia which is a ‘women in development’ project of Oxfam and AusAID. What they’re doing with simple technologies is working with Samoan families to produce organic virgin coconut oil which the Body Shop pays them seven times what you could get through local revenue. However if you don’t use anything organic, it’s off the list”.* (Government Minister, NZ)

Several key stakeholders believed that Samoa’s exports would not benefit significantly by the FTA, but that trade in services could become a major income-earner. However, this indirect economic gain could only be achieved with imports of funds being invested back into the Samoan economy via remittances. The implication of losing young workers to New Zealand in exchange for remittances was largely unquestioned.

*“The Recognised Seasonal Worker scheme could increase income by 40% (a real increase i.e. after accounting for money made if working in Samoa). People could work for up to 9 months of the year in New Zealand and earn remittances for their families back home”. *(CEO 3, NZ)

The potential for a specialised medical workers scheme to work in Samoa was mentioned by one stakeholder who believed that if you opened up specific areas to small foreign investments there would be the potential for medical specialists, including oncologists to work in Samoa. However, another stakeholder could not see how Samoa could provide incentives for specialised staff to work in Samoa when there are higher salaries abroad. The obvious tension between the two viewpoints underpins the faith invested in the promise and ideology of the global trade benefits.

Workforce development of medical and health workforce through training exchange programmes with Australia and New Zealand were also highlighted. This potential benefit relied on convincing the medical and health workforce in Samoa to participate ensuring these opportunities are included in a new agreement, is in itself a challenge.

*“The Doctors were a bit cynical … they said there have been arrangements in the past, exchange programs, but they didn’t last… (doctors should) give us the mechanics of how it works so that we can look at it and possibly include it”.* (Senior Advisor 1, Samoa)

Gender was a concern for some participants, who favoured agreements that would assist economic independence for women. Women were thought to be an under-recognised economic force within Samoa; trade agreements therefore needed to respond to women’s economic and political potential.

*“Gender is one of the main things that we look at, so that in any policy document that we have, including trade, we look at the role of women who comprise 50% or more of the (Samoan) population – women are more or less ignored and not taken into account”.* (Senior Advisor 2, Samoa)

Increasing the involvement of women in a range of areas including business, these improvements would be seen not only from an economic growth perspective for a country, but also within families which would see further flow-on effects for women’s wellbeing and also for other family members.

Most stakeholders agreed that the loss of tariffs through trade was a genuine concern for Pacific countries signing up to free trade deals. One stakeholder however, felt that regulatory changes prompted through trade agreements helped to counter revenue lost through tariffs:

“…we’ve had examples of countries that were sheltered and that had closed economies where tariffs were quite high and when it opened up with the liberalization programme, they collected more revenues – people reported stuff! No more under the table (dealings). It highlights the case where in terms of trade revenue, it has diversified – there are some issues, there are both pros and cons, but the benefits outweigh the costs”. (Business Consultant, Samoa)

### Trade: a double edged sword?

Many stakeholders were swift to make the link between trade and health consequences, most notably, regarding the impact on non-communicable diseases:

*“If Pacific countries continue to open their doors, could this spell bad news for Pacific countries in an already struggling attempt to address NCDs. Yes, without a doubt – what FTAs do is enable a breakdown in barriers to trade. The ability of Fiji and Samoa to restrict imports is severely limited. We saw that example when Fiji wanted to ban imports of lamb flaps and (the exporters) were going to take Fiji to WTO – it simply negates, eliminates and reduces the ability of island states to control”.* (CEO 2, NZ)

Another stakeholder made reference to the changing profile of affordable food choices within the Pacific, noting a shift from traditional crop-based nutrition to imported foods which are often highly-processed and energy-dense, low-nutritional foods:

*“Here is an article from the FAO on food security and changing diets in the Pacific which has a strong bearing on diets and NCD rates – this is becoming a serious issue for the Pacific region”.* (CEO 3, NZ)

One stakeholder, from the business sector, subscribed to the benefits of trade ideology, espousing the value of choice and individual responsibilities in the regulation of food choices.

*“The cost to health is dependent on lifestyle; people themselves, individual choice, just like smoking and drinking. Should it be a consideration of those negotiating trade? Well, access to products is made easier but you’ve got to be fully informed of implications of products, so have to have health standards in place to be able to ensure that consumers are well informed of implications on products”.* (Business Consultant, Samoa)

This perspective was later challenged by another stakeholder, who identified a fundamental flaw in the argument regarding individual responsibility and informed choice. (especially for Pacific Islands settings):

*“There is a huge knowledge deficit, a big difference between an informed consumer market and one that is uninformed. The second issue is that these populations don’t have the socioeconomic means to choose a healthier and more expensive option – they’re stuck with turkey tails, lamb flaps etc., they don’t have the economic power to choose”.* (CEO 2, NZ)

## Discussion

This study was undertaken to explore the perspectives of both New Zealand and Samoan stakeholders working in health or trade sectors, on the benefits and risks of FTA’s between the two countries. Our findings reveal evidence for both genuine concern and optimism regarding the potential impact of trade agreements on the health outcomes in Samoa. From the outset we acknowledge that our analysis is not fully representative of the views of either the New Zealand or Samoan governments about trade. Due to the small number of participants, our findings are likely to be only indicative of the range of opinions on this issue. Due to the small sample size, we are unable to include descriptors regarding the participants so to preserve their anonymity. Trade issues are highly sensitive in Samoa, which accounted for the small sample; few people were willing to or felt competent to discuss the trade issues in detail. Yet, our focused interviews uncovered a depth of concern about the reliance on trade as a mechanism for achieving sustainable health gains in Samoa. The critical importance of such an informed and engaging process of trade negotiations was questioned, particularly regarding the technical details embedded within the negotiation process and implications being presented during trade negotiations. Similarly, there was deep concern regarding the importance of ensuring new agreements included a tangible incentive, within a legal capacity, to support Samoa to determine and manage health risks as a result of free trade deals.

The benefits of participating in trade negotiations with New Zealand were explored and inevitably raised concerns for some and a determined hope for others. As anticipated, those from the trade or business sector expressed greater optimism about the potential for trade to raise economic standards, and therefore health outcomes in Samoa. Health advocates were less confident; past experiences have not borne the benefits promised, especially in vulnerable smaller economic jurisdictions [12]. These views perhaps signalled a need for greater investment in education and engagement around what trade means for small island developing nations, exposing where the opportunities may truly lie and where the risk and warning bells are indeed, ringing true. The benefits to health are also reflected by a review commissioned by Samoa to assess its development needs and constraints to inform trade negotiations for PACER Plus [13]. Another significant potential gain for Samoa is the potential for increasing women’s participation in employment such as, the Women in Business and Development Initiative in Apia. There is evidence to suggest that the benefits of empowering women to hold employment and to receive income have a possible flow-on effect for women’s wellbeing and for other members of her family which underpins development and health equity in low resourced settings such as Samoa.

For some stakeholders, the enthusiasm or sheer determination to see Samoa prosper appeared to override suggestions of negative implications for Samoa, particularly in respect to health outcomes. The intrinsic trust in the inherent value of free trade agreements for low and middle income countries, often illustrated through selective representation of success cases (e.g. China and India) is elevated due to the lack of comparable data on negative impacts. Migration further undermines development goals; promotion of off-shore employment inevitably contributes to the brain drain in the region. This is alongside the loss of tariffs incurred through signing of trade agreements and WTO accession. Tariffs are utilised by the Samoa government to inject funding into its health and education budgets and a loss would equate to a reduction in spend on these essential services for its people [14].

The massive increase in non-communicable diseases experienced across the Pacific has been attributed in part to a fundamental shift in lifestyles, notably in nutrition and employment [7]. Due to the binding nature of trade agreements, countries have little room to exert policy measures to protect and promote locally produced healthier food options. The rise in tobacco and alcohol consumption, which is largely imported, has devastating effects on the health of its citizens. The question of responsibility for trade implications on health variously shifted between those (business sector) stakeholders who prioritised the need for enhanced personal responsibility and those (predominantly health sector) who recognised the need for corporate or government responsibility in creating environments which are conducive to making healthy choices. As in most countries, the responsibility for health in Samoa is debated along a continuum of consumer choice at one end and local, national and/or international regulation at the other. It is expected that an increase in health promotion activities on healthy eating will ensure consumers make well-informed decisions; however health promotion activities will be conducted in an aggressive marketing environment by the makers of these products.

The process which Samoa follows in negotiating trade partnerships was for some informants consultative, informed and autonomous. For others, the process was influenced in part by the pressures from its trading partners and political factors such as the donor-recipient relationship between NZ and Samoa. Autonomy in itself is a measure of a country’s wellbeing, and trade policies that compromise its self-governance is viewed by some informants as a negative impact of trade relationships. Samoa’s recent lifting of its ban on turkey tails to meet WTO’s detailed criteria was cited by some as an example of this compromise. PACER Plus negotiations had mixed reviews among the informants; some were confident of a pro-development focus that will benefit Samoa and ensure improvement in health outcomes, while others were less certain of a consequence-free trade agreement between Samoa and NZ and Australia.

## Conclusion

Since Samoa’s accession to the World Trade Organisation in May 2012, even closer public scrutiny and rigorous research is needed to generate evidence of impact, not only on the growth in international investment, but on the health of its population. Careful consideration needs to be given to the rules of trade and the implications of signing trade agreements, particularly where decisions and compromises that initially create opportunities and wealth but inadvertently create inequalities and increased health and social disparities. Given the rapid and ever-changing landscape of trade, these actions should be taken sooner than later.

## Competing interests

The authors declare they have no competing interests.

## Authors’ contributions

JF, JMc and TP developed and refined the research concept, questions, methods; JF conducted all interviews, transcriptions and led the analysis. All authors read and approved the final manuscript.

## Pre-publication history

The pre-publication history for this paper can be accessed here:

http://www.biomedcentral.com/1471-2458/14/309/prepub
